# Saudi Arabia, pharmacists and COVID-19 pandemic

**DOI:** 10.1186/s40545-020-00243-1

**Published:** 2020-07-11

**Authors:** Ajaz Ahmad, Khalid M. Alkharfy, Ziyad Alrabiah, Abdulaziz Alhossan

**Affiliations:** Department of Clinical Pharmacy, College of Pharmacy, King Saud University, Riyadh, 11451 Saudi Arabia

**Keywords:** Saudi Arabia, Pharmacist, Covid-19, Pandemic, Pharmaceutical services

## Abstract

The latest outbreak of Covid-19 pandemic has placed a significant effect on health care system around the world. This article discusses the role of pharmacists in Saudi Arabia during the current Covid-19 pandemic. Pharmacists are an important part of everyday healthcare in Saudi Arabia. Pharmacists helped to protect the public from Covid-19 pandemic disease by participating in various initiatives including health education and promotion, medication dispensing, medication reconciliation, medication and patient counselling, training for self-management in current outbreak and emergency preparedness. Full utilization of skills of pharmacists boosted the safety response of Saudi Arabia to Covid-19 pandemic.

## Introduction

The COVID-19 was declared a Public Health Emergency of International Concern on 30 January 2020, and on 8 March 2020, the WHO declare the Covid-19 as a pandemic [1]. At this time, there are no specific vaccines or treatments for Covid-19. However, there are many ongoing clinical trials evaluating potential treatments. WHO continues to provide updated information as soon as clinical findings become available.

A total of 10,464,141 confirmed cases with 509,361 deaths linked to this pathogen as of June 30, 2020 have been reported [2]. Pharmacists played an important role in timely refilling of medications which reduced unnecessary hospital visits where individuals were at high risk of being exposed to Covid-19 [3]. Individuals or those undertaking home quarantine or isolation were provided home delivery service of medicines through community pharmacies [3]. Pharmacists recognized the incidence, dissemination and avoidance of spreading of Covid-19 pandemic. Apart from providing the pharmaceutical services, the pharmacy professionals were actively involved in community services like encouraging the public to wear surgical masks, maintaining social distance, usage of hand sanitizers and avoiding social gatherings.

The pharmacists were also participating in online webinars, following WHO guidelines on routine bases and discussing new strategies in combating the current pandemic. Pharmacist were performing patient counseling and drug reconciliation utilizing different interactive platforms during Covid-19 [4]. Strengthening pharmaceutical workforce can help to overcome COVID-19 and to achieve objectives of the Universal Health Coverage [5, 6].

Following are the measures to effectively utilize pharmacists’ expertise during the Covid 19 pandemic. Those involve clarifying pharmacist positions, coordinating and collaborating with pharmacists and maintaining capabilities for the workplace. Pandemic response from China, the USA, Canada, Japan and the UK are seen as indicators of how new regulations might be changed to enhance the practice of pharmacists through pandemic response [1].

## Saudi Arabia, COVID-19 pandemic and pharmacist

In Saudi Arabia, there are more than 190,800 reported cases of Covid-19 with 1649 deaths as of June 30, 2020 [2]. The first case of Covid-19 took place in Saudi Arabia on 2 March 2020. The outbreak led to temporary closure of air traffic, business establishments, government offices, educational institutions and public transport. People were no longer allowed to visit the two holy mosques in Makkah (for Umrah) and Almadinah [7]. The Kingdom stopped exporting all medical devices and products, including diagnostic or protective agents, to ensure their availability in Covid-19 pandemic. Many hospitals in different provinces were assigned in Saudi Arabia to treat Covid-19 incidents. The primary responsibility for handling a Covid-19 in Saudi Arabia lies with the Ministry of Health (MoH) and hospital organizations, each agreeing on the most appropriate approach to prepare and react.

The Kingdom of Saudi Arabia began to take many precautionary measures to combat the Covid-19. The immediate action was to set up a committee of various governmental organizations to determine and implement the actions needed against Covid-19 [8]. Since there is no vaccine available for the outbreak yet, avoidance is the only approach to protect the transmission of the virus; Consequently, the MoH and other departments carried out major initiatives to inform the public on various forms of stopping the transmission of the virus followed by the Ministry of Health (MoH) punch line campaign “We all are responsible”. These control measures played a significant role in restricting the transmission of SARS-CoV-2 with less mortalities. The mortality rate is very low in Saudi Arabia compared to other countries due to best health care facilities available at all levels in the Kingdom of Saudi Arabia [9].

During the time of public emergencies, the inclusion of pharmacists has helped to alleviate the burden on the other healthcare workers in health systems [10]. Pharmacists are the most accessible healthcare workers and thus understand the hardship of any outbreak like Covid-19 pandemic. They played a vital role in informing patients, encouraging the avoidance of diseases and referring any suspicious cases to suitable health care facilities in a timely manner [11]. Saudi Arabia has a well-equipped and advanced health system in the world [12], and improving use of medicines and medicines policy is an important part of healthcare [13]

Pharmacists are an important part of everyday healthcare in Saudi Arabia and have the ability to serve a number of positions during this latest Covid-19 pandemic [14]. Pharmacists are acknowledged for their importance in preventive care and in Covid-19 response; they stayed at the forefront for public safety by functioning as primary contact points.

The pharmacists in Saudi Arabia are providing a huge contribution by keeping patients safe at homes and provided them medications by mail as well as by offering continuous counseling remotely during Covid-19 [15]. During current Covid-19 crises, pharmacists in Saudi Arabia led to a broad delivery of medication management activity in clinical and non-clinical settings. In current Covid-19 crises, hospital pharmacists in Saudi Arabia formulated emergency medications, tracked and addressed product shortages, built remote pharmacy systems to avoid human-to-human infections, provided event-driven pharmaceutical treatment, informed the public on infection control and disease detection, helped in medication adherence, and counselled the patients for medication review and follow-up. Patients were being directed to use mobile applications for sending messages for required medications from pharmacies [16]. Several hospitals in Saudi Arabia have launched “drive-through pharmacy,” services allowing medicine to be picked up at the hospital so that there is no need for visitors to enter the premises. Once a doctor has prescribed the medications to a patient via a telephone or online consultation, the pharmaceutical team were notified. The hospital pharmacist then reviews the prescription order and ascertains the doses. Once the drug is ready, the pharmacist calls the patient to determine the best way of delivering the medicine. The means used for this pharmacy service included postal delivery and the drive-through pharmacy. Since the launch of the drive-through pharmacies, around thousands of patients were being serviced daily [17, 18]. Prevention and preparation for the Covid-19 pandemic are essential not only for the general public, but also for healthcare personnel in the clinical setting. Since the healthcare providers are at risk to get the disease, the hospital pharmacists encouraged the co-workers to wear personal protective equipment including gowns, goggles, face shields, facemasks and gloves.

Community pharmacists are the most available healthcare providers to the general population, so they have a great deal to say in responding to Covid-19 pandemic. This has led to significant changes in the health care systems of many countries [19, 20]. Community pharmacists in Saudi Arabia played a crucial role in proper medication usage that improved patient outcomes, and prevented misuse of medications. Their involvement has enhanced patient outcomes, quality of life, disease and drug knowledge and reduced utilization of health care services [21]. In Saudi Arabia, the community pharmacist role was not restricted to the pharmacy only but adequate reporting of suspected Covid-19 individuals to the concerned authorities. The community pharmacists in Saudi Arabia helped in the management of chronic illness, making sure that the medications are available and refilled, promoted continuous medication adherence and prescribed OTC medications which reduced the unnecessary hospital visits, where an individual is at high risk of being exposed to Covid-19. In Saudi Arabia the community pharmacies prepared information materials like posters, leaflets, app alerts and text messages in order to simplify the MOH guidelines related to the disease. Community pharmacies in Saudi Arabia also offered door to door step delivery of the medications, online counselling especially for the high-risk individuals and for those undertaken home quarantine or isolation. The pharmacists in Saudi Arabia played a key role in this endeavour. The eventual effect of the Covid-19 pandemic is likely to lead to a significant reorganization of global health care. It is necessary to keep up to date with current information about Covid-19, in order to tackle the containment of this pandemic [22, 23].
